# Enhanced adsorption of carbon sphere by doping with titania nanotubes for crystal violet removal: isotherm, kinetics, and thermodynamic studies†

**DOI:** 10.1039/d4ra04889j

**Published:** 2024-10-02

**Authors:** Ahmed M. E. Mohammed, Ahmed Kotb, Moustafa M. S. Sanad, Mohamed Abdel-Hakim, Abdelaal S. A. Ahmed

**Affiliations:** a Chemistry Department, Faculty of Science, Al-Azhar University Assiut 71524 Egypt ahmedkotb@azhar.edu.eg abdelaalsaiyd@azhar.edu.eg; b Central Metallurgical Research and Development Institute P.O. Box 87 Helwan 11421 Cairo Egypt

## Abstract

In this study, the carbon sphere (Cs) has been prepared and modified by titania nanotubes (TNTs) to be utilized as an adsorbent toward crystal violet (CV) dye as a model for cationic dyes from aqueous solution. The prepared TNTs@Cs composites has been characterized by various techniques such as XRD, SEM, and TEM analysis. The adsorption analysis displayed that the adsorption capacity of CV dye onto the modified Cs with TNTs is 92.5 mg g^−1^, which is much higher than that achieved by pristine Cs (12.5 mg g^−1^). Various factors that influence the overall adsorption processes, such as pH, contact time, initial CV dye concentration, adsorbent weight, and temperature, were studied. The TNTs@Cs_76.7_ composite showed the highest removal percentage of 99.00% at optimum conditions. The adsorption isotherm analysis showed that the experimental data of adsorption CV dye fitted better with the Langmuir isotherm model with *R*^2^ of 0.999, and the estimated maximum adsorption capacity was 84.7 mg g^−1^. On the other hand, the adsorption kinetic study showed that the adsorption of CV follows the pseudo-second order kinetic model with an equilibrium adsorption capacity (*q*_e_) of 10.66, 18.622, 47.61, and 48.31 mg g^−1^ for Cs, TNTs@Cs_93_, TNTs@Cs_86.8_, and TNTs@Cs_76.7_ composites, respectively. The thermodynamic analysis showed negative free energy (Δ*G*) values, this indicates that the adsorption of CV is a spontaneous and feasible process. Furthermore, the Δ*H* and Δ*S* are positive values that indicate an endothermic adsorption process. Furthermore, the prepared TNTs@Cs_76.7_ composite displayed remarkable adsorption stability and the removal efficiency of CV remains at 96.3% after five cycles.

## Abstract 2

In this study, the carbon sphere (Cs) has been prepared and modified by titania nanotubes (TNTs) to be utilized as an adsorbent toward crystal violet (CV) dye as a model for cationic dyes from aqueous solution.

## Graphical abstract

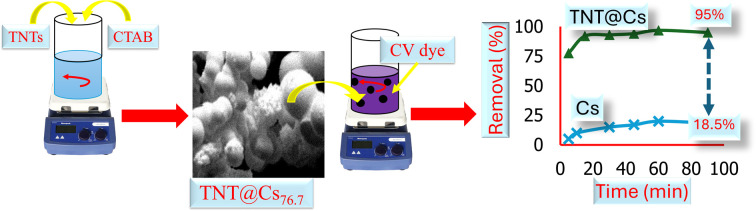

## 1. Introduction

A growing freshwater problem is being experienced globally as a result of the fast increase in the world's population, the effects of climate change, and industrial development on water quality.^1^ Considering this, various consumers and polluters of freshwater significantly contribute to freshwater depletion. The most significant sources of industrial pollutants come from a variety of industries, including the textile, cosmetic, leather, food, pharmaceutical, paint and varnish, and pulp and paper industries. Among them are the increasingly used dyes, such as methylene blue (MB), rhodamine B (RhB), methyl orange (MO), Congo red (CR), Disperse Violet 26, methyl red, and crystal violet (CV).^2^ It is estimated that, about 700 000–1 000 000 dyes annually produced through a variety of industrial processes, including food processing, pharmaceutical, textile, paper, rubber, plastic, and cosmetics industries.^3^ From this it has been reported that every year between 40 000 and 50 000 tons of dyes are dumped into the water systems.^4^ These dyes harm the aquatic ecosystem by lowering the aesthetic value of water features and obstructing light penetration.^5^ Some dyes have toxic, mutagenic properties, and can even cause cancer when combined with their metabolites. These dyes also cause problems for human renal, liver, and nervous system dysfunction. They are difficult to decolorize due to their complicated structure.^6,7^ Thus, there is a great interest from the environmentalists and researchers to remove these harmful compounds from wastewater.^2,8,9^

CV is a member of the triphenylmethane group that is widely used in veterinary and animal medicine as a biological stain, to identify bloody fingerprints, and in various industrial textile processes.^10^ Furthermore, in certain concentrations, CV dye can result in several ailments and diseases, including cyanosis, cancer, mutagenesis, respiratory failure, eye irritation, elevated heart rate, and skin irritation. Since CV dye is stable and resistant to microbial degradation, it needs to be removed from wastewaters prior to their discharge in order to maintain environmental safety.^11^ Till now, there are various techniques such as disorientation, electrochemical, coagulation, ozonation, nano filtration, reverse osmosis, advance oxidation processes, photocatalytic degradation, and adsorption are used to eliminate such these pollutants from wastewater.^8,9,12–15^ Among all these techniques, the adsorption processes are most commonly where it is cost-effective, highly efficient, and economic feasibility.^16,17^ The main issue facing adsorption process is the adsorbent materials and their morphologies which plays a crucial role in overall adsorption performance.^18,19^ In the last decades, a lot of materials have been used as effective adsorbent materials toward the organic dyes such as carbons,^16,20,21^ metal oxides,^22^ bio-polymer-based composites.^23^ Because of their exceptional stability, distinctive structure, and abundance of raw material sources, carbon materials have found widespread application in a variety of industries.^24^ Among the many benefits of carbon spheres (Cs) in particular are its uniform shape, adjustable particle size distribution, and customized porosity. Consequently, research has been conducted on them for various applications such as adsorption,^25^ catalysis,^26^ energy conversion and storage,^27^ biomedical,^28^ and environmental.^29^

There are various methods for production of carbon spheres such as emulsion polymerization,^30^ hydrothermal carbonization (HTC),^31^ soft and hard templating,^32^ and self-assembly.^33^ A significant advance was made in 2011 by Liu *et al.*,^34^ who extended the Stober method to manufacture carbon spheres with adjustable particle sizes. This approach has gained a lot of interest due to its high yield, easy to follow operating procedures, and inexpensive and accessible precursor. Recently, Farbod, and Sharif,^35^ prepared Cs by hydrothermal method toward methylene blue (MB) dye, and Congo red (CR) dye adsorption. However, the adsorption of CV toward dyes is limited, thus its import needs to modify the Cs to be effective adsorbent. Due to their enormous surface area and distinctive tubular architectures, a cheap, readily accessible, non-toxic, and thermally stable substance, TiO_2_ nanotubes (TNTs) could be an excellent additive to improve the adsorptive property of Cs to remove organic contaminants from water streams.^36^ In this work, we prepared TNTs using a hydrothermal process at low calcination temperature, and then added to Cs framework in different portions, and the obtained TNTs@Cs composites were employed to remove CV dye from aqueous solution. The influence of different parameters such as solution pH, contact time, temperature, initial CV dye concentration and adsorbent dosage on adsorption efficiency were studied. The outcomes show that TNTs@Cs composites displayed outstanding reusability and adsorption performance. The synthesis and application of a novel adsorbent material for the removal of cationic contaminants from water are highlighted in this work. As a result, the material offers a wide range of possible applications in the future for wastewater purification.

## 2. Experimental

### 2.1. Materials

Titanium(iv) oxide (titanium dioxide (GPR)) (98%, ADWIC), sodium hydroxide (NaOH, 96%, ADWIC), hydrochloric acid (HCl, 33%, PIOCHEM), glucose (d (+) glucose monohydrate, 99.5%, Biotech), CTAB (cetyltrimethylammonium bromide, 100%, ALDRICH), crystal violet dye (Alpha Chemika). The used solutions were prepared by using distilled water (GFL, Germany).

### 2.2. Preparation of adsorbent materials

#### 2.2.1. Preparation of carbon sphere

Exactly, 4.52 g of glucose and 0.018 g of CTAB were dissolved in 45 mL of distilled water. The solution reacted at 200 °C for 6 h, then the product was subjected to centrifugation (3500 rpm, 5 min). The obtained solid product was washed many times with distilled water, and finally dried at 100 °C for 12 h.^37^

#### 2.2.2. Preparation of TNTs

Exactly 0.762 g of titanium dioxide (TiO_2_) was disperssed in 63 mL NaOH solution (10 M), then transferred in 100 mL Teflon – lined loaded in the stainless-steel autoclave and placed in a furnace at 130 °C for 24 h. After that, the product was separated by centrifuge (3500 rpm, 5 min) and washed many times with distilled water until the pH reached ≈8. Then, the product was treated with 500 mL of 0.1 M HCl overnight and washed again many times with distilled water until the pH reached ≈6.5, and finally dried overnight at 80 °C.^38^

#### 2.2.3. Preparation of TNTs@Cs composites

A series of TNTs@Cs composites using different mass ratios of glucose was prepared. Typically, a certain amount of glucose and 0.018 g of CTAB were dissolved in 45 mL of distilled water (see Table 1) and kept under ultrasonication for 0.5 h. On the above solution, 0.343 g of TNTs was added and then mixture was kept at magnetic stirrer for 0.5 h, followed by ultrasonication for another 0.5 h. Then, the solution heated at 200 °C under hydrothermal conditions for 6 h to complete the reaction. The obtained product was separated by centrifuge (3500 rpm, 5 min), washed many times with distilled water until the pH reached ≈5.5 and dried at 100 °C for 12 h.

**Table 1:** The various amount of glucose in grams used for preparing TNTs@Cs composites

Amount of glucose (g)	Amount of CTAB (g)	Volume of water (mL)	Composite
4.52	0.018	45 mL	TNTs@Cs_93_
2.26			TNTs@Cs_86.8_
1.13			TNTs@Cs_76.7_

### 2.3. Batch adsorption studies

The removal of CV dye from an aqueous solution was investigated in a batch system. All the adsorption experiments were done in a 100 mL beaker with a magnetic stirrer at a constant speed of 200 rpm. The effect of different process parameters such as solution pH (2–7), time (5–90 min), initial dye concentration (10–100 mg L^−1^), adsorbent dosage (5–70 mg/25 mL), and temperature (20–50 °C) on CV dye removal was investigated. The remaining concentration of CV dye was measured by a UV-visible spectrophotometer at a wavelength of 580 nm. The percentage removal of CV dye and equilibrium adsorption amount of CV dye *q*_e_ (mg g^−1^) were calculated by using the following equations. The percentage removal of CV dye was calculated by eqn (1).1
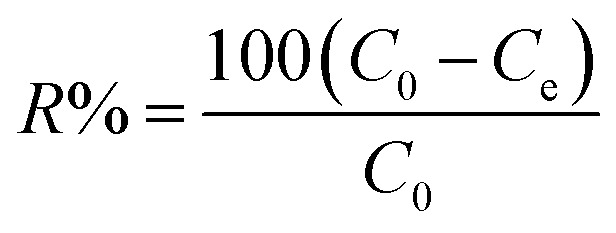


Adsorption amount of CV dye per gram of adsorbent (mg g^−1^) was determined by eqn (2).2
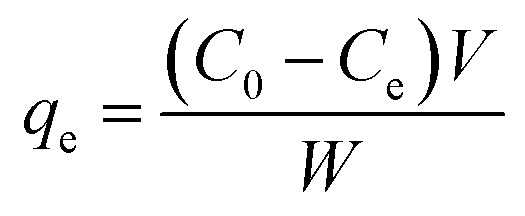
where *C*_0_ is the initial concentration of CV (mg L^−1^), *C*_e_ is the equilibrium concentration of CV dye (mg L^−1^), *V* is the volume of solution (L), and *W* is the mass of the adsorbent (g).

## 3. Results and discussion

### 3.1. XRD results

**Fig. 1:**
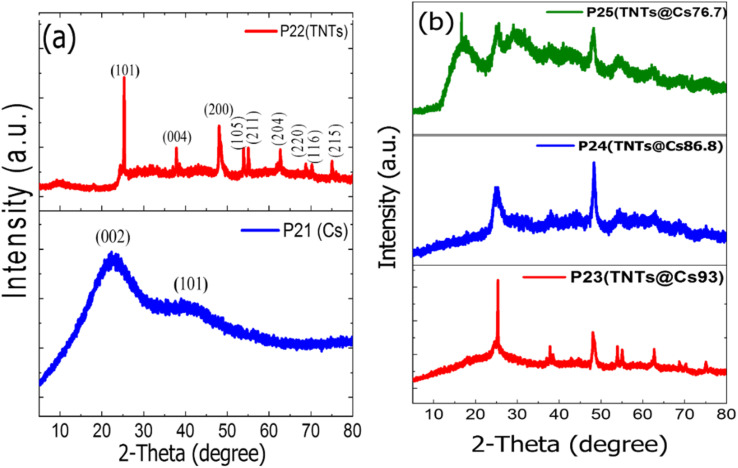
XRD patterns of (a) Cs, and TNTs. (b) TNTs@Cs_93_, TNTs@Cs_86.8_ and TNTs@Cs_76.7_ composites.

The crystalline structure of the samples was determined by X-Ray Diffraction (XRD). As shown in Fig. 1a two broad peaks at ∼21° and ∼42.5° are detected for Cs, which corresponding to the (002) and (101) diffractions of graphitic carbon, respectively.^39^ The characteristic diffraction peaks of TNTs located at 25.281°, 37.801°, 48.050°, 53.891°, 55.062°, 62.690°, 68.762°, 70.311° and 75.032° corresponding to the diffraction patterns of (101), (004), (200), (105), (211), (204), (116), (220), and (215) anatase planes, respectively (JCPDS 21-1272). The characteristic diffraction peaks of TNTs@Cs_93_ composite are almost the same as TNTs peaks (Fig. 1b). It was noted that the characteristic reflections of TNTs became weaker and broader with decreasing amount of glucose precursor, illustrating the reduction of crystallinity degree and crystal size as shown in the case of TNTs@Cs_86.8_ and TNTs@Cs_76.7_ composites, especially at TNTs@Cs_76.7_ composite that shows a broad carbon peak at 16.6°. The crystallite size of the TNTs@Cs composites are determined by the Scherrer equation (eqn (3)).3
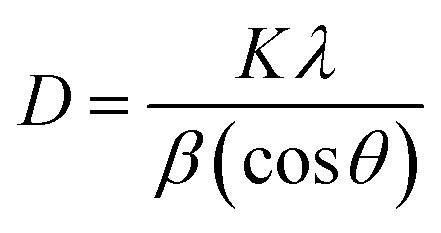
where, *D* is the crystallite size in nm, *λ* is the wavelength of the radiation (0.154056 nm), *K* is a constant equal to 0.9, *β* is the peak width at half-maximum intensity and *θ* is the peak position.

Scherrer equation shows that crystallite size of TNTs@Cs_76.7_ composite is the smallest as shown in Table 2. This feature is also in agreement with the observations in the TEM images.

**Table 2:** Crystalline size of TNTs, and TNTs@Cs composite by Scherrer equation

Adsorbent	TNTs	TNTs@Cs_93_	TNTs@Cs_86.8_	TNTs@Cs_76.7_
Crystallites size (nm)	32.5	32.5	9.5	7.6

### 3.2. BET analysis

**Fig. 2:**
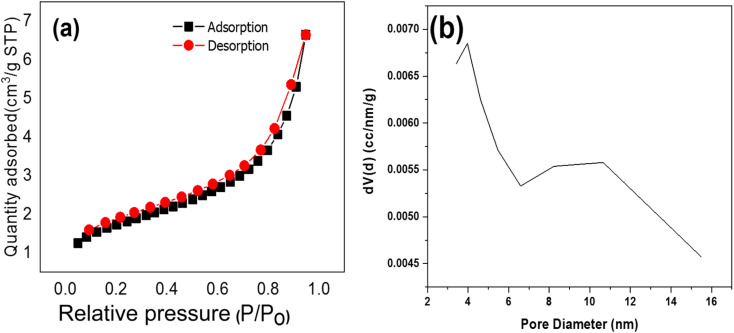
(a) N_2_ adsorption–desorption isotherms, and (b) the pore size distribution of TNTs@Cs_76.7_ composite.

The effects of pore size, pore volume, and specific surface area of TNTs@Cs_76.7_ composite is essential in the adsorption process. The adsorption isotherms profiles for the TNTs@Cs_76.7_ composite is a type IV isotherms, which are corresponding to mesoporous material as shown in Fig. 2. Table 3 shows the BET measurements of TNTs@Cs_76.7_ composite. The specific surface area (m^2^ g^−1^) and pore size distribution of the adsorbent were estimated by the Brunauer–Emmett–Teller (BET) equation in the partial pressure range from 0.05 to 0.30 and the Barrett–Joyner–Halenda (BJH) model from the desorption branch of the isotherms, respectively. The specific surface area of TNTs@Cs_76.7_ composite (≈79.042 m^2^ g^−1^). TNTs@Cs_76.7_ composite has a pore size of 3.966 nm and a pore volume of 0.082 cm^3^ g^−1^. The increased surface area of TNTs@Cs_76.7_ composite can improve the surface activity and surface reaction of composite material.^40^

**Table 3:** The BET parameters of TNTs@Cs_76.7_ composite

Material	Surface area	Pore volume (cm^3^ g^−1^)	Pore size (nm)
TNTs@Cs_76.7_	79.042	0.082	3.966

### 3.3. SEM analysis

**Fig. 3:**
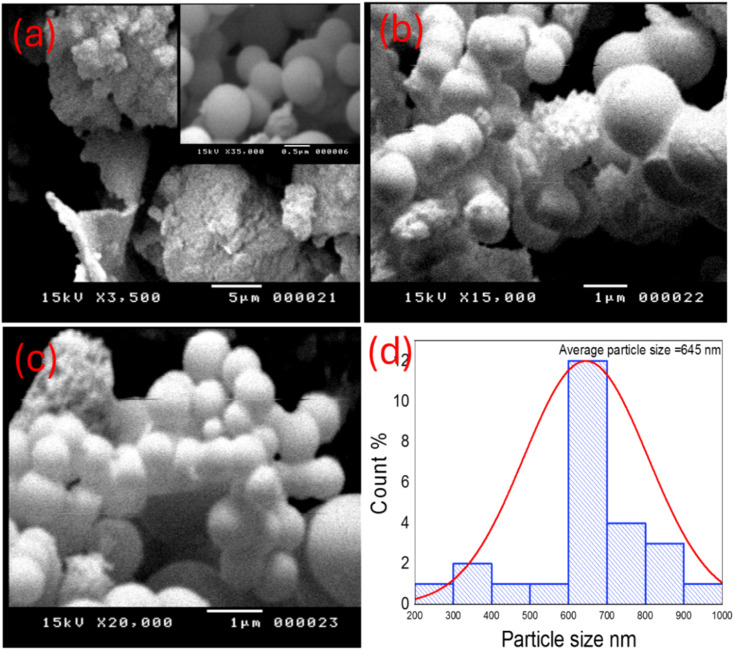
(a–c) SEM images, and (d) histogram of particle size of TNTs@Cs_76.7_ composite.

The scanning electron microscope (SEM) was employed to evaluate the surface morphology of TNTs@Cs_76.7_ composite as shown in Fig. 3 (a–c). The composite shows a clear predominate appearance of spherical carbon with the presence of some irregular and other sheet like vertically oriented parts. It could be seen that the average size of Cs is about 645 nm, as shown in Fig. 3d. The size of spheres in SEM analysis was higher than that of TEM analysis due to inconsistent calibration of magnification, secondary electron emission increase, and sample preparation.^41^

### 3.4. TEM analysis

**Fig. 4:**
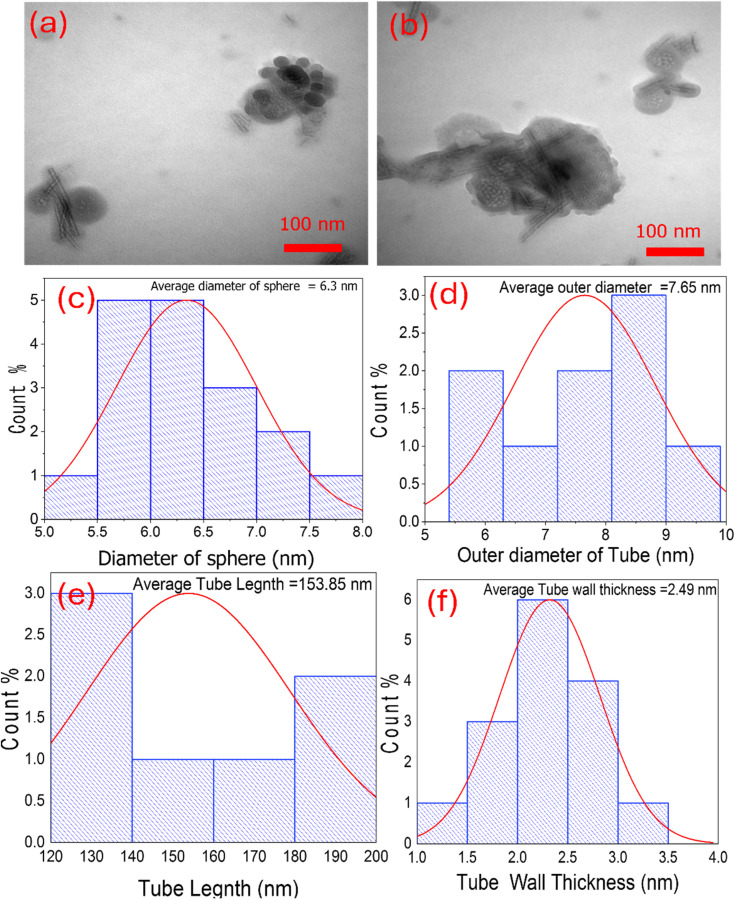
(a and b) TEM images of TNTs@Cs_76.7_ composite. Histograms represent (c) the diameter of sphere, (d) the outer diameter of tube, (e) tube length, and (f) tube wall thickness of TNTs@Cs_76.7_ composite.

The morphology features and microstructure were further investigated by transmission electron microscopy (TEM). Fig. 4 depicts the TEM results of the TNTs@Cs_76.7_ composite. It was shown that the intercalation of tubular structures and spheres can be observed clearly. The average diameter of the sphere was about 6.3 nm (Fig. 4c). The TNTs with a uniform inner diameter of about 4.8 nm and an outer diameter of about 7.6 nm, where as the thickness of the tube wall was about 2.5 nm as observed in Fig. 4d. The length of the nanotubes ranges from about 120–200 nm and the average length is estimated to be 153.9 nm.

### 3.5. XPS analysis

**Fig. 5:**
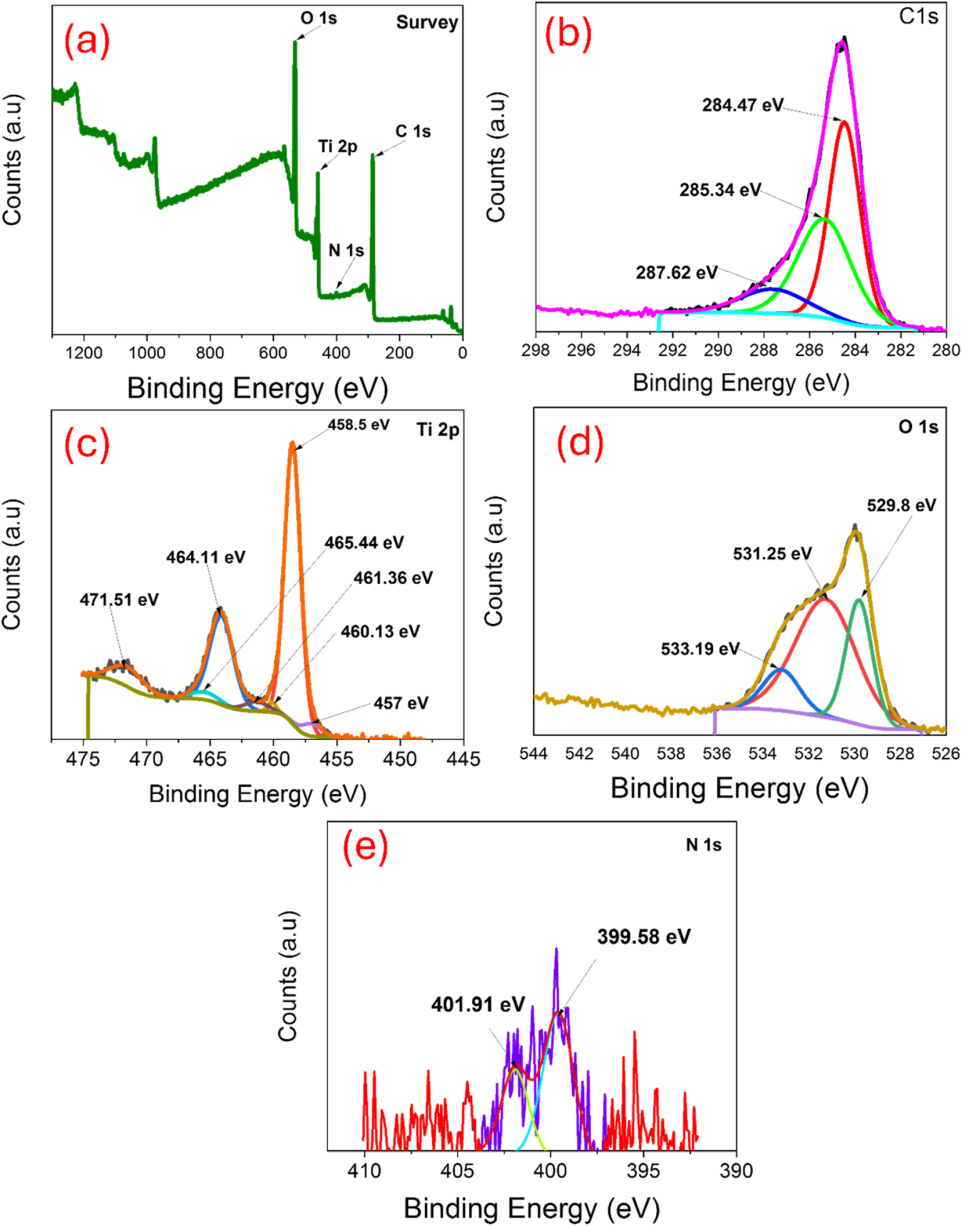
XPS of TNTs@Cs_76.7_ composite: (a) survey, (b) C 1s, (c) Ti 2p, (d) O 1s, and (e) N 1s.

To determine the chemical composition and valence states of all the elements on the surface, TNTs@Cs_76.7_ composite was investigated by X-ray photoelectron spectroscopy. All XPS profiles were deconvoluted into some sub-peaks and shown in Fig. 5.

As represented in Fig. 5a, the full XPS survey spectrum of TNTs@Cs_76.7_ composed of C, O, Ti, and a small percentage of N. The binding energies for C 1s, O 1s, Ti 2p and N 1s were 285.81, 531.64, 459.23 and 400.9 eV respectively. Fig. 5b revealed the C 1s XPS curve fitted in three peaks at 284.47, 285.34, and 287.62 eV were attributed to C–C, (C–H/C–O), and (C–O–C/C–O) bonds, respectively.^42^ The electron binding energies correspond to the spin orbits of Ti 2p_3/2_ at 458.5 eV, and Ti 2p_1/2_ at 464.11 eV (Fig. 5c). These binding energy values are consistent with the Ti^4+^ chemical state, which can prove that the Ti element exists in the TiO_2_ lattice as well as in the Ti^4+^ chemical state. In O 1s XPS spectrum (Fig. 5d), three peaks at 529.8, 531. 25 and 533.19 eV were related to Ti–O–Ti, C=O, and C–*O*–C/C–OH bonds, respectively.^43^ In N 1s XPS spectrum (Fig. 5e), two peaks at 399.58, and 401.91 eV were related to benzenoidamine(–NH–) and graphitic N (N–C_3_), respectively.^44^

### 3.6. Zeta potential measurement

**Fig. 6:**
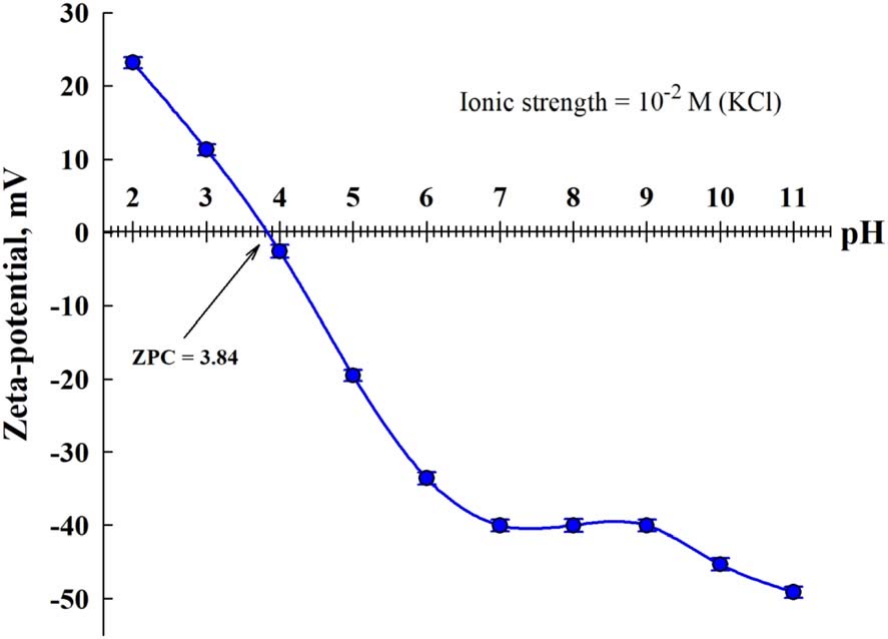
Zeta potential TNTs@Cs_76.7_ composite before CV adsorption.

The point of zero charge (pH_PZC_) of TNTs@Cs_76.7_ composite before CV adsorption was measured by zeta potential test at room temperature to forecast the charge on the surface of the adsorbent.^45^Fig. 6 showed the zeta potential of TNTs@Cs_76.7_ composite *versus* the pH of the solution.

The PZC for TNTs@Cs_76.7_ composite is estimated to be 3.84. At pH values less than pH_PZC_ the surface of the TNTs@Cs_76.7_ composite possess a positive charge, whereas, at higher pH value, the surface charge of TNTs@Cs_76.7_ composite was negative which cations may be adsorbed. Due to its cationic nature, the CV dye exhibits stronger electrostatic interactions with the negatively charged TNTs@Cs_76.7_ composite at a pH higher than 4.

### 3.7. Batch adsorption of CV dye

#### 3.7.1. Effect of pH

**Fig. 7:**
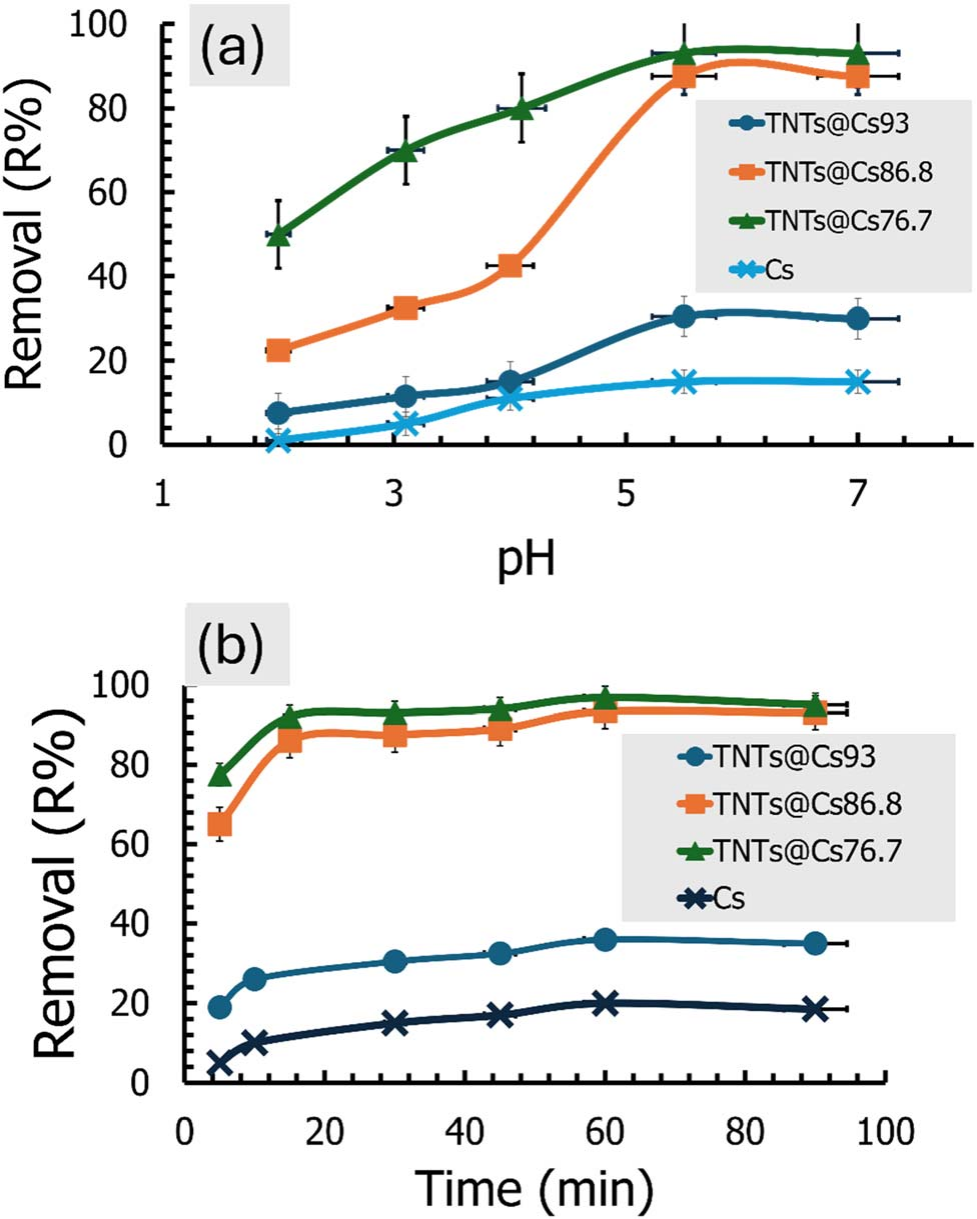
(a) Effect of pH, and (b) effect of contact time on the adsorption performance of CV under optimized conditions.

The adsorptive ability of the adsorbent is highly influenced by the solution pH, especially in an acidic medium from pH (2–5.5) then remain constant at neutral medium, where the maximum removal was found at pH 5.5. As presented in Fig. 7a, at alkaline medium, CV dye concentration is reduced without adding adsorbent, in strongly basic solutions, the purple monovalent CV^+^ cation slowly combines with hydroxide ions and forms a neutral colorless product (CV–OH) as shown in eqn (4).^46^4
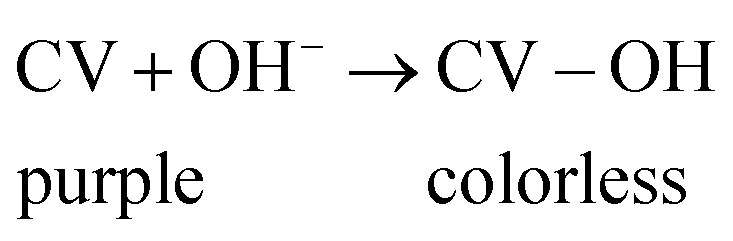


So, pH was investigated from pH (2 : 7) as shown in Fig. 7a.

#### 3.7.2. Effect of contact time

The effect of time interval on CV adsorption using Cs, TNTs@Cs_93_, TNTs@Cs_86.8_, and TNTs@Cs_76.7_ composites was investigated at 5, 15, 30, 45, 60, and 90 min (*C*_0_: 20 ppm; adsorbent dose: 0.01 g; pH: 5.5; temperature: 293 K; agitation speed: 200 rpm). The results in Fig. 7b revealed that CV adsorption at the initial period is very fast due to the many free active sites for TNTs@Cs_86.8_ and TNTs@Cs_76.7_ composites. Over time, the adsorption process slows down so that the adsorption percentage becomes parallel to the *x*-axis, which is considered almost constant at 15 min. Fig. 7b represents the removal efficiency of Cs, TNTs@Cs_93_, TNTs@Cs_86.8_ and TNTs@Cs_76.7_ composites at an optimized time. Maximum adsorption is obtained by Cs (20%), TNTs@Cs_93_ (36%), TNTs@Cs_86.8_ (93.4%) and TNTs@Cs_76.7_ (96.8%) at 60 min, which confirms that it is optimized contact time.

#### 3.7.3. Effect of initial CV dye concentrations

**Fig. 8:**
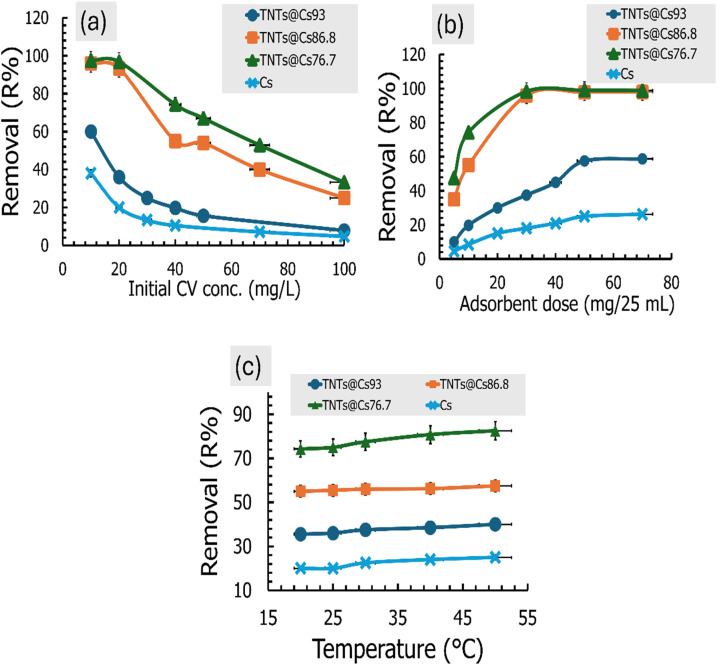
(a) Effect of the initial dye concentration, (b) effect of the adsorbent dosage, and (c) effect of the temperature on the CV adsorption performance.

The effect of dye concentration on CV adsorption using Cs, TNTs@Cs_93_, TNTs@Cs_86.8_ and TNTs@Cs_76.7_ composites was investigated at 10, 20, 40, 50, 70, and 100 mg L^−1^. The adsorbent process was performed at room temperature (298 K), and the pH of the CV solution was adjusted to 5.5. Then 0.01 g of adsorbent was added and the mixture was kept under a magnetic stirrer at 200 rpm for 1 h. As shown in Fig. 8a, the percentage removal of the CV was decreased exponentially with the increase in initial concentration. By increasing the initial concentration from 10 to 100 mg L^−1^, the percentage removal decreased from 38% to 4.8%, 60% to 7.8%, 96% to 25% and 97.5% to 33.25% for Cs, TNTs@Cs_93_, TNTs@Cs_86.8_ and TNTs@Cs_76.7_ composites, respectively. This may be due to lack of available active sites required for the high initial concentration of CV.

#### 3.7.4. Effect of adsorbent dose

The effect of dosage on adsorption of CV was investigated by various weights (5, 10, 30, 50, and 70 mg) of Cs, TNTs@Cs_93_, TNTs@Cs_86.8_ and TNTs@Cs_76.7_ composites. The initial concentration of CV dye was fixed at 40 mg L^−1^, the pH was adjusted at 5.5, and the mixture was stirred with agitation speed of 200 rpm at room temperature for 1 h. Fig. 8b represents the removal efficiency of Cs, TNTs@Cs_93_, TNTs@Cs_86.8_ and TNTs@Cs_76.7_ composites at optimized adsorbent dosage. It was detected that with an increase in the adsorbent weight, removal of CV dye was enhanced at the initial stage as more active charge bearing sites were available for the adsorption. Hence, after the saturation point, no further increase in adsorption was recorded, although the quantity of adsorbent increases. At higher dosages, 50 and 70 mg/25 ml, the adsorption efficiency was almost the same. Maximum adsorption is obtained by Cs (26.25%), TNTs@Cs_93_ (58.75%), TNTs@Cs_86.8_ (98%) and TNTs@Cs_76.7_ (99%) at 70 mg, which confirms the best adsorbent dose for CV dye.

#### 3.7.5. Effect of temperature

Temperature has a vital role in deciding the adsorption process whether it is either endothermic or exothermic in nature. Therefore, it is needed to find the optimum temperature to get the best adsorption efficiency. In the present study, the effect of temperature on the removal efficiency of CV dye was investigated at temperature range (20–50 °C). The obtained results are shown in Fig. 8c. It was observed that the percentage removal of CV was slightly increased with an increase in the temperature from 20 to 50 °C, indicating the process is endothermic in nature.

### 3.8. Adsorption kinetics

The kinetic behaviors of CV dye adsorption by Cs, TNTs@Cs_93_, TNTs@Cs_86.8_ and TNTs@Cs_76.7_ composites were investigated. Three well-known adsorption kinetic models in linear and non-linear forms were applied. We stated bellow the linear form of these models; the pseudo-first order (PFO) eqn (5),^47^ and pseudo second order (PSO) eqn (6).^48^

5 ln(*q*_e_ − *q*_t_) = ln *q*_e_ − *K*_1_*t*

6
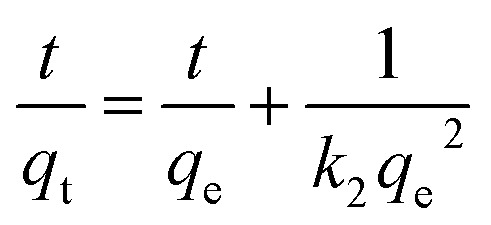


The rate-limiting step is difficult to determine by the PFO, and the PSO models. To solve this issue, an intraparticle diffusion (IPD) model has been used, the linear form are expressed by eqn (7).^49^

7 *q*_t_ = *k*_p_*t*^0.5^ + *C*

where *q*_e_ and *q*_t_ are the amounts of adsorbed dye in mg g^−1^ at equilibrium and time *t*, respectively. *k*_1_ (min^−1^), *k*_2_ (mg g^−1^ min^−1^), and *k*_p_ (mg g^−1^ min^−1^) are the PFO, PSO, and IPD rate constants respectively. *C* (mg g^−1^) is the thickness of the porous medium.

**Fig. 9:**
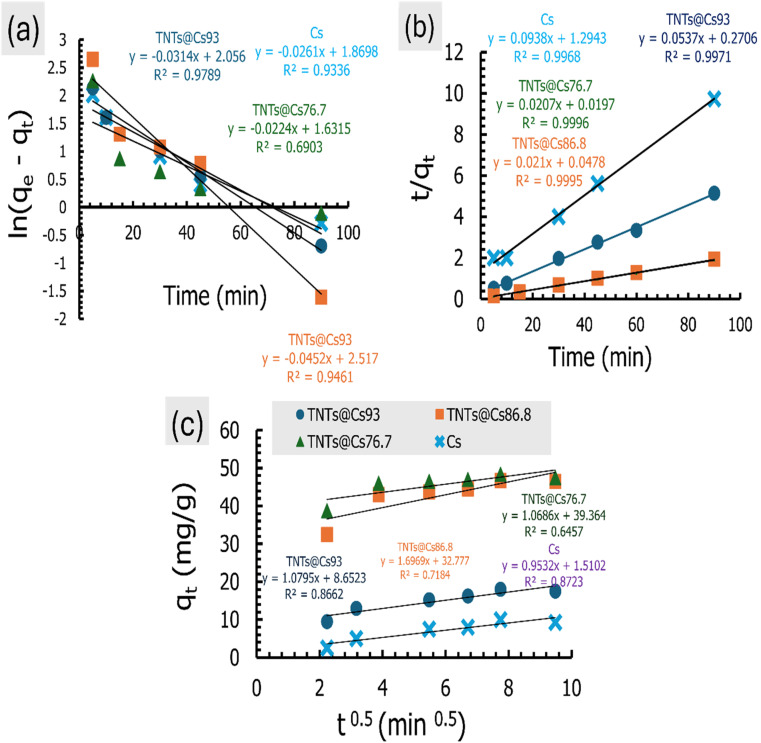
The linear adsorption kinetics for the (a) PFO, (b) PSO, and (c) IPD models.

The linear kinetic models for the adsorption experimental work of CV onto Cs, TNTs@Cs_93_, TNTs@Cs_86.8_ and TNTs@Cs_76.7_ composites are presented in Fig. 9, and their estimated kinetic parameters listed in Table 4. While the non-linear kinetic fitting models, and their related parameters are in ESI data file (Fig. S1 and Table S1).† It has been observed that the experimental work of adsorption CV is best fitted with linear models, which is typically obtained by the best correlation coefficient (*R*^2^) value. From the linear models, the experimental data of adsorption CV onto the Cs, TNTs@Cs_93_, TNTs@Cs_86.8_ and TNTs@Cs_76.7_ composites are best fitted with the PSO kinetic models, as indicated by higher *R*^2^ values. The *q*_e_ values were estimated to be 10 and 18, 46.7, and 48.4 mg g^−1^ for the adsorption of CV dye by Cs, TNTs@Cs_93_, TNTs@Cs_86.8_ and TNTs@Cs_76.7_ composites, respectively. This indicates the adsorption of CV dye is a chemical adsorption process.^50^

**Table 4:** The parameters of the linear kinetic models for the adsorption of CV removal

Adsorbent	*q*_e,exp_ (mg g^−1^)	Pseudo first order			Pseudo second order			Intraparticle diffusion		
		*q*_e,cal_ (mg g^−1^)	*K*_1_ (1/min)	*R* ^2^	*q*_e,cal_ (mg g^−1^)	*k*_2_ (mg g^−1^ min^−1^)	*R* ^2^	*C* (mg g^−1^)	*k*_p_ (mg g^−1^ min^−1^)	*R* ^2^
Cs	10	6.5	0.0261	0.934	10.66	0.0068	0.997	1.510	0.953	0.872
TNTs@Cs_93_	18	7.8	0.0314	0.979	18.622	0.0106	0.997	8.652	1.080	0.866
TNTs@Cs_86.8_	46.7	12.4	0.0452	0.946	47.61	0.0092	0.999	32.777	1.697	0.718
TNTs@Cs_76.7_	48.4	5.1	0.0224	0.690	48.31	0.0217	0.999	39.364	1.069	0.646

The kinetic behaviour of CV dye was also investigated in relation to the IPD model, and the plotted data are showed in Fig. 9c, and its parameters are depicted in Table 4. This plot should be a straight line passing through the origin for adsorption systems where IPD is the governing mechanism. This indicates that the adsorption rate at any given time should be linearly proportionate to the square root of the time. As in Fig. 9c, the fact that the straight line does not pass through the origin suggests that there are other controls for adsorption rate besides the IPD process.^51^ A certain amount of boundary layer management is required for the adsorption process, and extragranular diffusion processes including liquid film diffusion and surface adsorption also have an impact on the adsorption rate.^52^

### 3.9. Adsorption isotherms

**Fig. 10:**
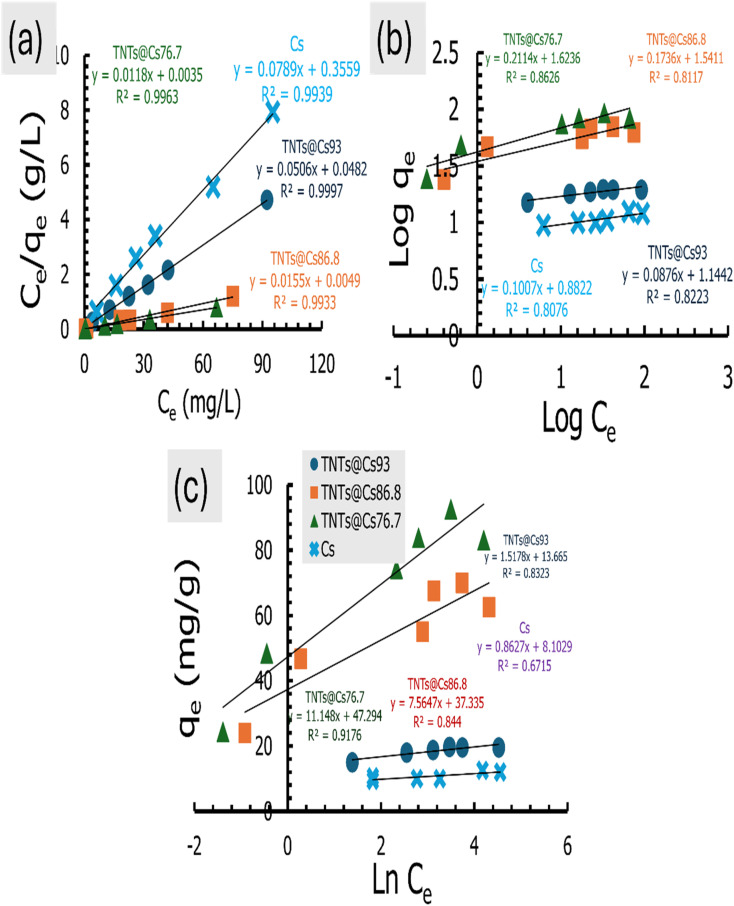
(a) Langmuir, (b) Freundlich, and (c) Temkin isotherm for CV dye adsorption.

The adsorption isotherm is necessary for explaining adsorbent–adsorbate interactions to express the adsorption capacity.^48^ Here, the Langmuir, Freundlich, and Temkin isotherm models in both linear and non-linear forms were employed to investigate the adsorption of CV dye. The estimated linear isotherm curves are presented in Fig. 10, and their related parameters are in Table 5. The non-linear curves and estimated data are in the ESI data (Fig. S2 and Table S2).† It can be observed from Tables 5 and S2 (ESI data),† the adsorption isotherms of CV dye onto the Cs, TNTs@Cs_93_, TNTs@Cs_86.8_ and TNTs@Cs_76.7_ composites are fitted well with linear isotherm models as indicated by higher correlation coefficient (*R*^2^). Thus, in the next lines we will discuss only the data estimated from the linear adsorption isotherm modes.

**Table 5:** The parameters estimated from the linear adsorption isotherm models for the adsorption of CV dye

Adsorbent	Freundlich model			Langmuir model			Temkin model			
	*K*_f_ (mg g^−1^)	1/*n*	*R* ^2^	*K*_L_ (L mg^−1^)	*q*_m_ (mg g^−1^)	*R* ^2^	*R*_L_	*b*_t_	*A*_t_ (L g^−1^)	*R* ^2^
Cs	7.625	0.1007	0.808	0.2217	12.6742	0.994	0.043	2823.7	11 997.921	0.672
TNTs@Cs_93_	13.938	0.0876	0.882	1.0498	19.7628	0.999	0.009	1604.9	8128.750	0.832
TNTs@Cs_86.8_	34.76	0.1736	0.811	3.1633	64.5161	0.993	0.003	322.02	139.132	0.844
TNTs@Cs_76.7_	42.035	0.2114	0.863	3.3714	84.7458	0.996	0.003	218.51	69.573	0.918

The Langmuir isotherm model is used for describing monolayer adsorption on adsorbent surfaces with a limited number of active sites.^48,53^ According to this model, all the sites on the adsorbent surface are energetically equal. Furthermore, this model presupposes that adsorbed molecules do not interact laterally. The linear form of Langmuir isotherm is expressed by eqn (8).^54^8
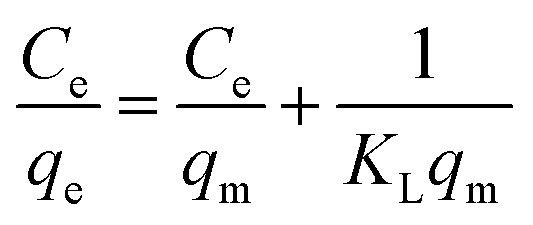
where *C*_e_ (mg L^−1^) is the dye equilibrium concentration, *q*_e_ (mg g^−1^) is the quantity of dye adsorbed at equilibrium, *q*_m_ (mg g^−1^) is the maximum adsorbent capacity, and *K*_L_ (L mg^−1^) is Langmuir constant, which is connected to the adsorption energy.

The linear Langmuir plots of the experimental data are presented in Fig. 10a, and the related parameters are in Table 5. As presented in Table 5, the Langmuir model afforded a relatively higher correlation coefficient (*R*^2^) among other investigated isotherm models for Cs, TNTs@Cs_93_, TNTs@Cs_86.8_ and TNTs@Cs_76.7_ composites with an estimated maximum adsorption capacity of 12.67, 19.76, 64.52, and 84.74 mg g^−1^, respectively.

The important factor of the Langmuir isotherm is the separation factor (*R*_L_), which is dimensionless quantity, and can be calculated by eqn (9).9
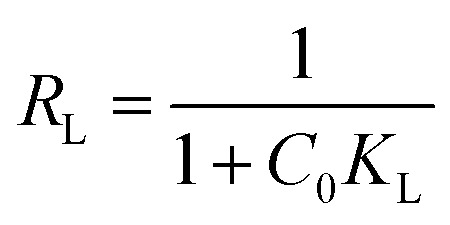


The *R*_L_ value indicates whether it is linear (*R*_L_ = 1), irreversible (*R*_L_ = 0), favorable (0< *R*_L_ <1) or unfavorable (*R*_L_ >1).

The estimated *R*_L_ values are 0.043, 0.009, 0.003, and 0.003 for Cs, TNTs@Cs_93_, TNTs@Cs_86.8_ and TNTs@Cs_76.7_ composites, respectively. All in belowl, which depicts that the adsorption of CV dye on our materials are favorable.

From the above discussion, the Langmuir model is suitable for predicting the adsorbent–adsorbent interactions, as well as the adsorption capacities of the prepared materials for CV dye at relatively high.

The Freundlich adsorption isotherm is considered an empirical equation for a heterogeneous system in which multilayer adsorption may occur on the adsorbent surface.^55^ The Freundlich plots are shown in Fig. 10b, and the related parameters are in Table 5. The Freundlich isotherm is stated as in eqn (10).10
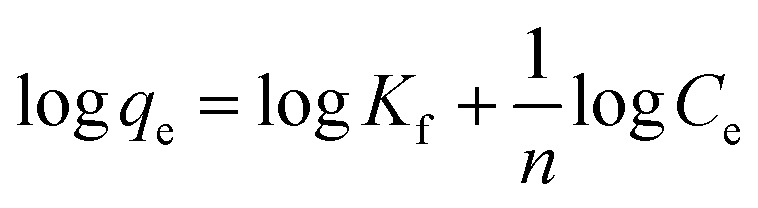
where 1/*n*, and *K*_f_ are constants that are used to characterise the adsorption capacity and intensity, respectively. The value of *n*, for favorable adsorption should be in between 0.1–1.

As presented in Table 5, the lower *R*^2^ value for the Freundlich model as compared to Langmuir isotherm indicates the absence of multilayer physisorption. Moreover, the value of 1/*n* below one and closer to 0 indicates a favorable and heterogeneous. This study shows that the Freundlich model is unsuitable for predicting the absorbent–adsorbent interactions and the relative adsorption capacities of the prepared materials for CV dye.

Temkin isotherm model is usually used to investigate the effects of adsorbate–adsorbate interactions on the adsorption process.^56^ Only an intermediate range of ion concentrations is covered by the Temkin isotherm.^57^ The equation for Temkin isotherm is expressed as eqn (11).

11 *q*_e_ = *B*_T_ ln(*C*_e_) + *B*_T_ ln *A*_T_

Where,12
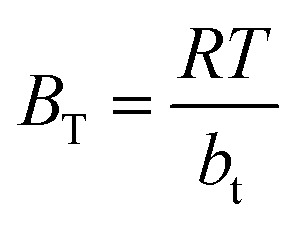
where *A*_T_ (L g^−1^) is the equilibrium binding constant, *B*_T_ (J mol^−1^) is constant related to heat of sorption associated with the parameter *b*_t_ as Temkin isotherm constant, *R* is the universal constant of gases (8.314 J mol^−1^ K), and *T* is the absolute temperature (K).

The values of the constants are calculated from the slope and intercept of the plot in Fig. 10c, which is *q*_e_*vs.* ln *C*_e_. The values of both *B*_T_ and *A*_T_ are presented in Table 5. It has been reported that the typical adsorption energies, (*B*_T_ ln(*A*_T_)), for a chemisorption in the range of 8–16 kJ mol^−1^, and *b*_t_ values are higher than 80 kJ mol^−1^.^58^ From the slopes, and intercepts in Fig. 10c, the estimated values of *B*_T_ ln(*A*_T_) for Cs, TNTs@Cs_93_, TNTs@Cs_86.8_ and TNTs@Cs_76.7_ composites are 8.102, 13.665, 37.335, and 47.294 kJ mol^−1^, respectively. The corresponding *b*_t_ values are 2.872, 1.632, 0.328, and 0.222 kJ mol^−1^, respectively. This results illustrate that the interactions between CV dye and the surfaces of Cs, TNTs@Cs_93_, TNTs@Cs_86.8_ and TNTs@Cs_76.7_ composites are weak, indicating that the adsorption processes are physical adsorption.^59^

### 3.10. Thermodynamic investigation

The effect of temperature on the adsorptions of CV on Cs, TNTs@Cs_93_, TNTs@Cs_86.8_ and TNTs@Cs_76.7_ composites was examined, and the thermodynamics of this process was examined to understand the comprehension of the adsorption behaviors. The thermodynamic parameters, including the enthalpy (Δ*H*^°^), entropy (Δ*S*^°^) and Gibbs free energy (Δ*G*^°^) were evaluated (eqn (13)–(16)).

13 Δ*G*° = −*RT* ln *K*

14
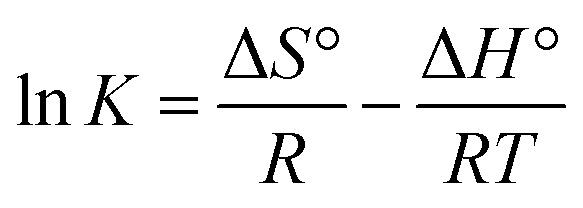
15
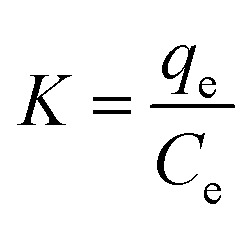

16 Δ*G*° = Δ*H*° − *T*Δ*S*°

**Fig. 11:**
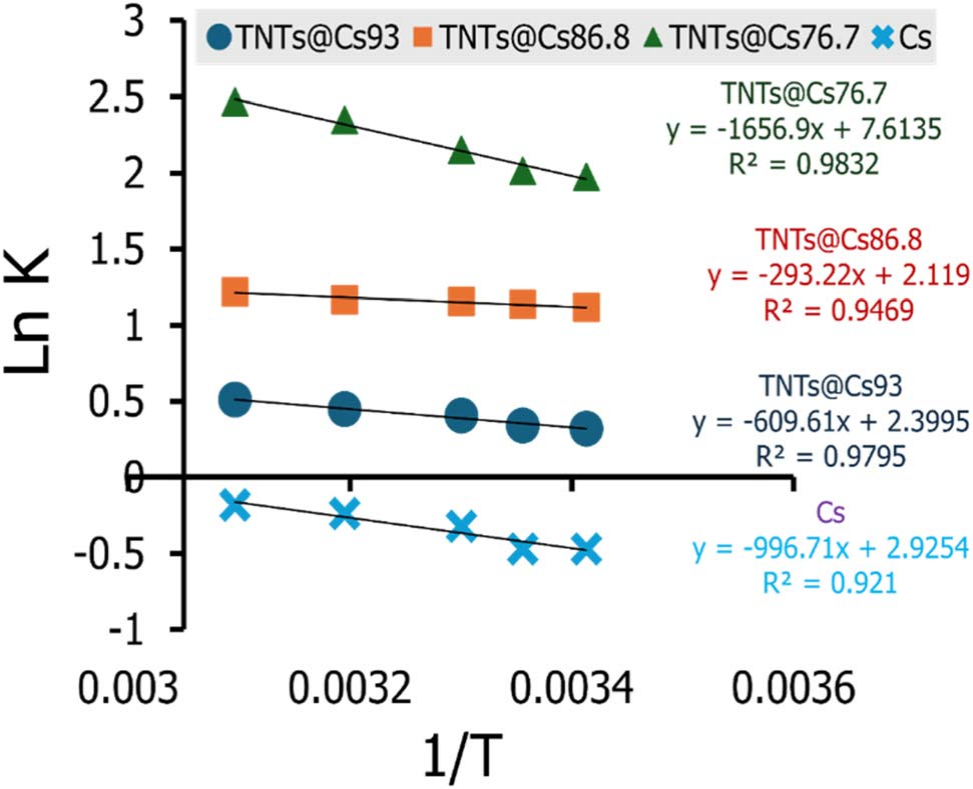
The linear relation of van't Hoff equation at various temperature from 293 to 323 K for adsorption of CV.

The Δ*G*°, Δ*H*°, and Δ*S*° (kJ mol^−1^) were calculated from the plot of ln *K vs.* 1/*T* as shown in Fig. 11, and the thermodynamic parameters were monitored in Table 6. The positive value of Δ*H*° and Δ*S*° exhibited that the adsorption process of CV dye onto Cs, TNTs@Cs_93_, TNTs@Cs_86.8_, and TNTs@Cs_76.7_ composites were endothermic in nature.^60^ This means that the adsorption efficiency may be enhanced by increasing the solution's temperature. This also confirms that the adsorption is chemically processed. The negative values of Δ*G*° for (TNTs@Cs_93_, TNTs@Cs_86.8_, and TNTs@Cs_76.7_) adsorbents at all temperatures indicated that the process of adsorption in the present work is spontaneous and feasible, except the Cs adsorbent which Δ*G*° has positive value that indicated that the process of adsorption is not spontaneous.^61^

**Table 6:** Thermodynamic parameters of CV dye

Adsorbent	*T*(K)	Δ*G*° (kJ mol^−1^)	Δ*H*° (kJ mol^−1^)	Δ*S*° (kJ mol^−1^ K)
Cs	298	0.476	3.801	0.011
	303	0.421		
	313	0.309		
	323	0.198		
	393	−0.583		
TNTs@Cs_93_	298	−0.402	2.325	0.009
	303	−0.448		
	313	−0.539		
	323	−0.631		
	393	−1.272		
TNTs@Cs_86.8_	298	−1.291	1.118	0.008
	303	−1.330		
	313	−1.411		
	323	−1.492		
	393	−2.058		
TNTs@Cs_76.7_	298	−2.334	6.319	0.029
	303	−2.479		
	313	−2.769		
	323	−3.059		
	393	−5.092		

### 3.11. Reusability

**Fig. 12:**
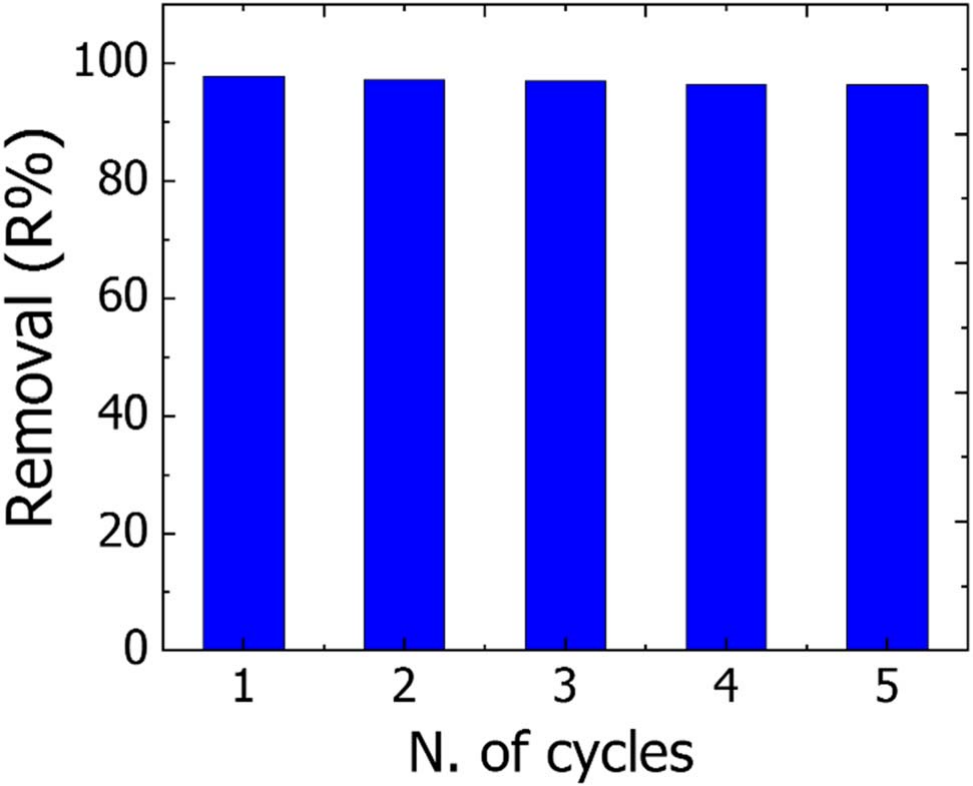
Regeneration of the TNT@Cs_76.7_ composite up to five successive desorption–adsorption cycles.

To reduce the overall cost of the water treatment process and apply adsorbent on a large scale/industrial scale, a reusability test on the adsorbent materials must be carried out.^16,62^ Here the reusability of the TNT@Cs_76.7_ composite that displayed the best overall removal performance among our prepared adsorbents was investigated by monitoring their adsorption toward CV dye under ideal conditions for five cycles. First, the adsorption process of CV onto the TNT@Cs_76.7_ composite was performed by mixing 0.06 g TNT@Cs_76.7_ composite with 25 mL of CV dye with an initial concentration of 40 ppm, and stirred for 30 min at room temperature. For the regeneration, the CV adsorbed onto TNT@Cs_76.7_ composite was sonicated repeatably two times with 20 ml ethanol for 30 min, and then washing two times with distilled water, and finally subjected to drying in an oven to be used for the next cycle. After desorption, the regenerated TNT@Cs_76.7_ composite was reused for CV dye adsorption, and five cycles of regeneration and adsorption were carried out in succession. As shown in Fig. 12, the removal percentage of CV dye was observed at 97.75% in the first cycle, reaching 96.3%. This indicates that it can remove organic dyes over several cycles.

### 3.12. Adsorption mechanism of CV dye by TNT@Cs_76.7_ composite

**Fig. 13:**
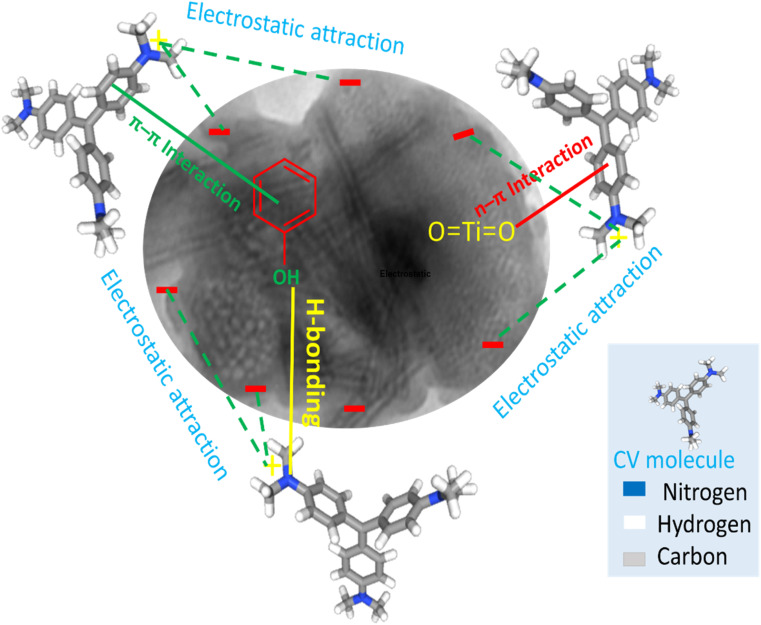
Adsorption mechanism of CV dye by TNT@Cs_76.7_ composite.

As discussed above, the mechanism of adsorption of CV dyes onto TNT@Cs_76.7_ composite can be described as follow. According to zeta potential, the pH_ZPC_ of TNT@Cs_76.7_ composite surfaces were performed at pH 3.84. Thus, at pH higher than pH_PZC_ the surface of the TNTs@Cs_76.7_ composite possess a negative charge, and thus the cationic dye is absorbed on the adsorbent surface due to the electrostatic force between negative charge of adsorbent surface and positive charge of CV dye in basic conditions.^63^ H-bonding can occur between the H-donor (nitrogen in CV dye) and the H-acceptor groups (OH groups in the TNTs@Cs_76.7_ composite). Another factor that can affect the adsorption mechanism is the n–π interaction between the oxygen (electron-donating) on the TiO_2_ adsorbent surface and the π-system in the aromatic rings of the dye molecules (electron acceptor).^64^ In addition, π^+^–π interaction is another factor for CV adsorption on the TNTs@Cs_76.7_ composite. The CV as organocation has amine groups that can act as π electron acceptors and involve with the formation of π^+^–π interaction with the π electron-rich polyaromatic surface of Cs. Hence, the electron-rich Cs surface of the TNTs@Cs_76.7_ composite can bind with the protonated amino group of the CV dye forming π^+^–π electron donor–acceptor interactions. Thus, as described in Fig. 13, the adsorption mechanism of CV dye with TNTs@Cs_76.7_ composite has been controlled by electrostatic attraction, H-bonding, n–π electron donor–acceptor, and π^+^–π electron donor–acceptor interactions.

### 3.13. Interference of co-existing dyes on the removal of CV dye

**Fig. 14:**
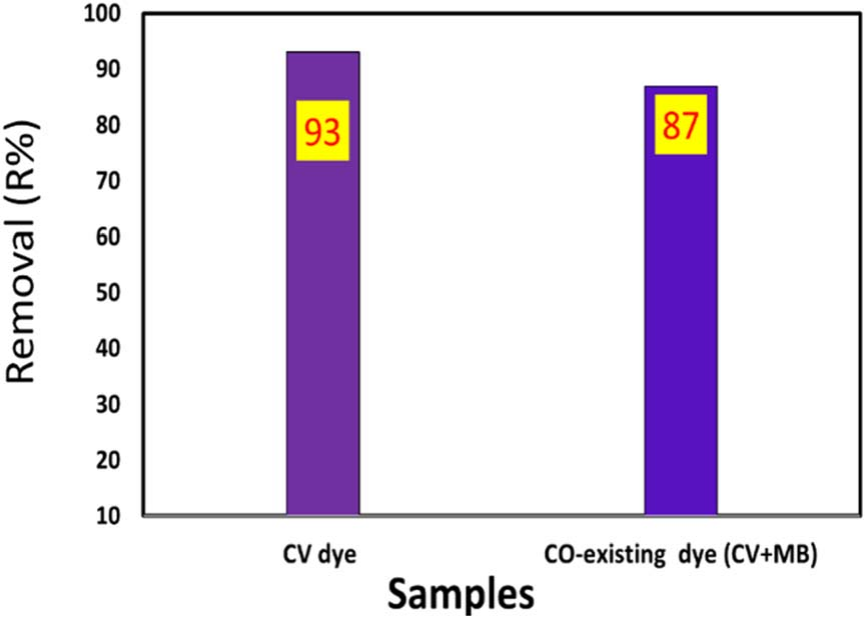
Effect of co-existing MB dye with CV on the adsorption efficiency by TNT@Cs_76.7_ composite.

To evaluate the effect of co-existing dyes on the removal efficiency of TNT@Cs_76.7_ composite toward CV, we add 25 mL of CV and MB dye mixture in a 4 : 1 (v/v) ratio to 0.01 g of TNT@Cs_76.7_ composite. The initial concentrations of CV and MB dyes are 20 ppm, and the TNT@Cs_76.7_ composite weight was fixed at 0.01 g. The adsorption was carried out under magnetic stirring at room temperature for 30 min. As presented in Fig. 14, by adding 25% of MB onto CV dye, the TNT@Cs_76.7_ composite displayed little decreasing in adsorption affinity toward CV.

### 3.14. The practical applicability of the prepared adsorbent

**Fig. 15:**
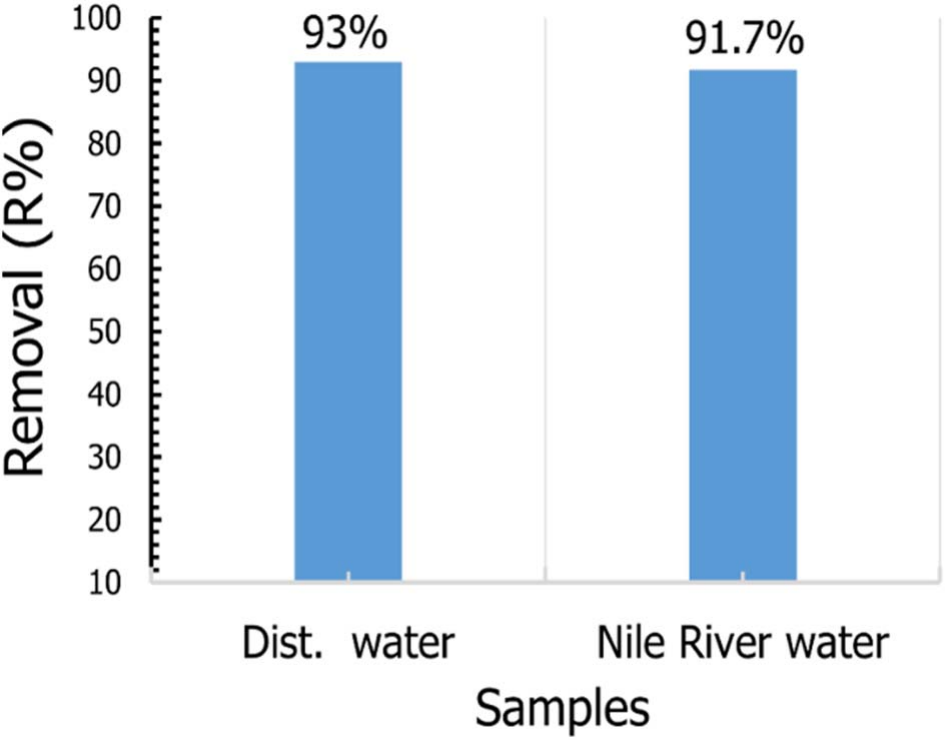
Effect of TNT@Cs_76.7_ composite toward CV prepared by dist. Water, and Nile River water samples.

To study the applicability of our prepared materials for removal of CV dye from the real water sample, we use TNT@Cs_76.7_ composite toward removal of CV dye solution prepared by Nile water as a real sample with a condition presented in Table 7. To compare the removal efficiency of TNT@Cs_76.7_ composite, we also prepare CV dye solution with distillated water under the same conditions. The adsorption experiment was performed by mixing 25 ml CV dye in a concentration of 20 ppm with 0.01 g of TNT@Cs_76.7_ composite and stirred for 30 min at room temperature. The removal percentage of CV from the real sample was found to be 91.7%, which is very close to that prepared for wastewater as shown in Fig. 15.

**Table 7:** The analysis of utilized Nile River water sample

Element	Concentration	Element	Concentration
TSD (ppm)	170	Chlorides (ppm)	20
Turbidity (NTU)	4	Calcium hardness (ppm)	66
Total alkalinity (ppm)	160	Magnesium hardness (ppm)	44
Total hardness (ppm)	110	Ammonia (ppm)	0.1
		Iron (ppm)	0.1
		Manganese (ppm)	0.05

### 3.15. Comparison of adsorbent with other reported adsorbents

According to the literature, several carbon sphere-based materials have been utilized for the removal of CV dye from aqueous solutions. The competitiveness of our absorbent was examined against the other reported adsorbents, as illustrated in Table 8. Our adsorbents showed competitive adsorption ability and affinity towards CV dye concerning the other adsorbents. Hence, TNT@Cs_76.7_ composite is highly recommended as efficient adsorbents for cationic dyes.

**Table 8:** Comparison of removal of dyes by our adsorbents and similar reported adsorbents

Adsorbent	Dye	Conditions	*q*_max_ (mg g^−1^)	Ref.
Hollow carbon spheres/graphene hybrid aerogels	MO	—	344.1	65
	Rhodamine B (RhB)		441.5	
Activated carbon spheres from discarded maize cobs	Rose bengal dye (RB)	pH = 3, time = 60 min	303.6	66
Carbon spheres	MB	—	15.4	67
Hierarchically porous graphitic carbon spheres	MB	—	182.8	
Modified carbon spheres	Paraquat (PQ)	pH = 6, time = 180 min	134.63	24
	Chrysoidine G (CG)	pH = 7, time = 600 min	299.44	
	Malachite green (MG)	pH = 6, time = 720 min	326.93	
Chemically activated carbon spheres	MB	pH = 7, time = 180 min	602.4	68
TNTs@Cs_76.7_	CV	pH 5.5; 60 min	84.7	Our work

## 4. Conclusion

In this study, an efficient adsorbent TNTs@Cs composites were prepared from glucose and Titania for remediation wastewater from organic hazardous materials such as CV dye. TNTs@Cs composites was characterized using, XRD, SEM, TEM, XPS and N_2_ adsorption desorption isotherm analysis. TEM analysis showed that TNTs@Cs composites consist of spheres and tubular structures. Results revealed that adsorption of CV dye onto TNTs@Cs composites strongly controlled by pH, contact time, dye concentration, adsorbent weight and temperature. The quantity of dye uptake (mg g^−1^) was found to rise with increase in dye concentration but reduced with rise in adsorbent dose. The pH 5.5 was found to be the optimum pH for dye removal. The results obtained under the optimum conditions showed that, TNTs@Cs_76.7_ composite has considerable removal percentage of 99.00%. The adsorption equilibrium behavior of CV was best fitted with Langmuir isotherm model with maximum adsorption capacities of 84.7 mg g^−1^. The adsorption of CV follows the pseudo-second order kinetic model. In addition, thermodynamic study exhibited the adsorption of CV dye onto TNTs@Cs_76.7_ composite was spontaneous and endothermic in nature. Also, TNTs@Cs_76.7_ composite displayed high stable adsorption performance with a removal percentage of 96.3% after five cycles.

## Data availability

All data have been included in the main manuscript, and in the ESI data file.†

## Author contributions

Ahmed M. E. Mohamed: experimental work, and writing – original draft. Abdelaal S. A. Ahmed design the methodology, supervision the adsorption process, editing, and full revision the manuscript, Ahmed Kotb, material suggestion, and supervision of the synthesis materials. Moustafa M. S. Sanad performing the physical characterizations in his working institute. Mohamed Abdel-Hakim is the leader supervision of the study.

## Conflicts of interest

The authors declare that they have no known competing financial interests or personal relationships that could have appeared to influence the work reported in this paper.

## Supplementary Material

RA-014-D4RA04889J-s001
