# Supplementary material for: Enhanced adsorption of carbon sphere by doping with titania nanotubes for crystal violet removal: isotherm, kinetics, and thermodynamic studies
Source: RSC Adv. 2024 Oct 2;14(43):31332–47. doi: 10.1039/d4ra04889j (PMC11445704; doi:10.1039/d4ra04889j)
Supplement: RA-014-D4RA04889J-s001 [file RA-014-D4RA04889J-s001.pdf]

Supplementary Information (SI) for RSC Advances.
This journal is © The Royal Society of Chemistry 2024

Supplementary data

Enhanced adsorption of carbon sphere by doping with titania nanotubes for crystal violet removal: Isotherm, kinetics, and thermodynamic studies

Ahmed M. E. Mohamed[1], Ahmed Kotb[1], Mohamed Abdel-Hakim[1], Moustafa M. S Sanad [2], Abdelaal S. A. Ahmed[1*]

[1] Chemistry Department, Faculty of Science, Al-Azhar University, Assiut 71524, Egypt

[2] Central Metallurgical Research and Development Institute, P.O. Box 87 Helwan, 11421, Cairo, Egypt

Correspondence: abdelaalsaiyd@azhar.edu.eg (A. S. A. Ahmed)

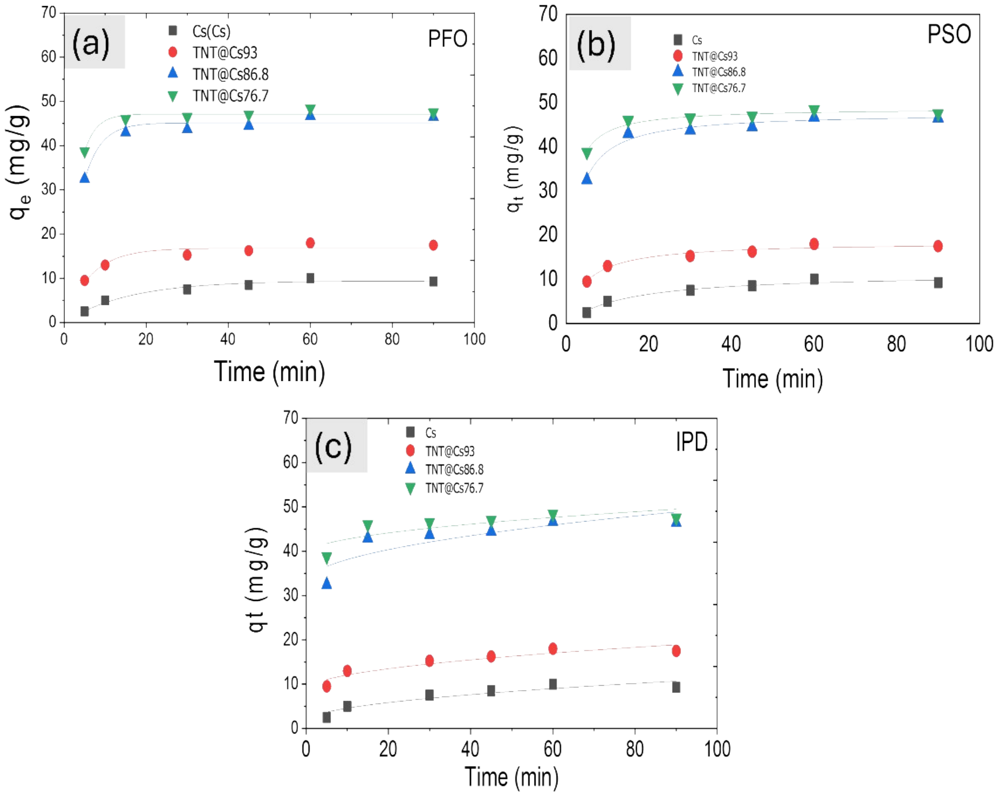

Figure S1: The non-linear kinetics fitting curves for the (a) PFO, (b) PSO, and (c) IPD models.

Table S1: The parameters estimated from the non-linear adsorption kinetic models for the adsorption of CV dye.

**Adsorbent**	**Pseudo first order**			**Pseudo second order**			**Intraparticle diffusion**		
	$q_{e,cal}$ **(mg/g)**	K_1 **(1/min)**	R^2	$q_{e,cal}$ **(mg/g)**	k_2 **(mgg^{-1}/min)**	R^2	**C (mg/g)**	k_p **(mgg^{-1}/min)**	R^2
**Cs**	9.42	0.064	0.957	11.38	0.006	0.958	1.52	0.965	0.834
**TNTs@Cs$_{93}$**	16.82	0.156	0.892	18.42	0.012	0.959	8.65	1.08	0.833
**TNTs@Cs$_{86.8}$**	45.15	0.250	0.929	47.73	0.009	0.962	32.78	1.70	0.648
**TNTs@Cs$_{76.7}$**	47.15	0.343	0.941	48.74	0.017	0.957	39.36	1.068	0.557

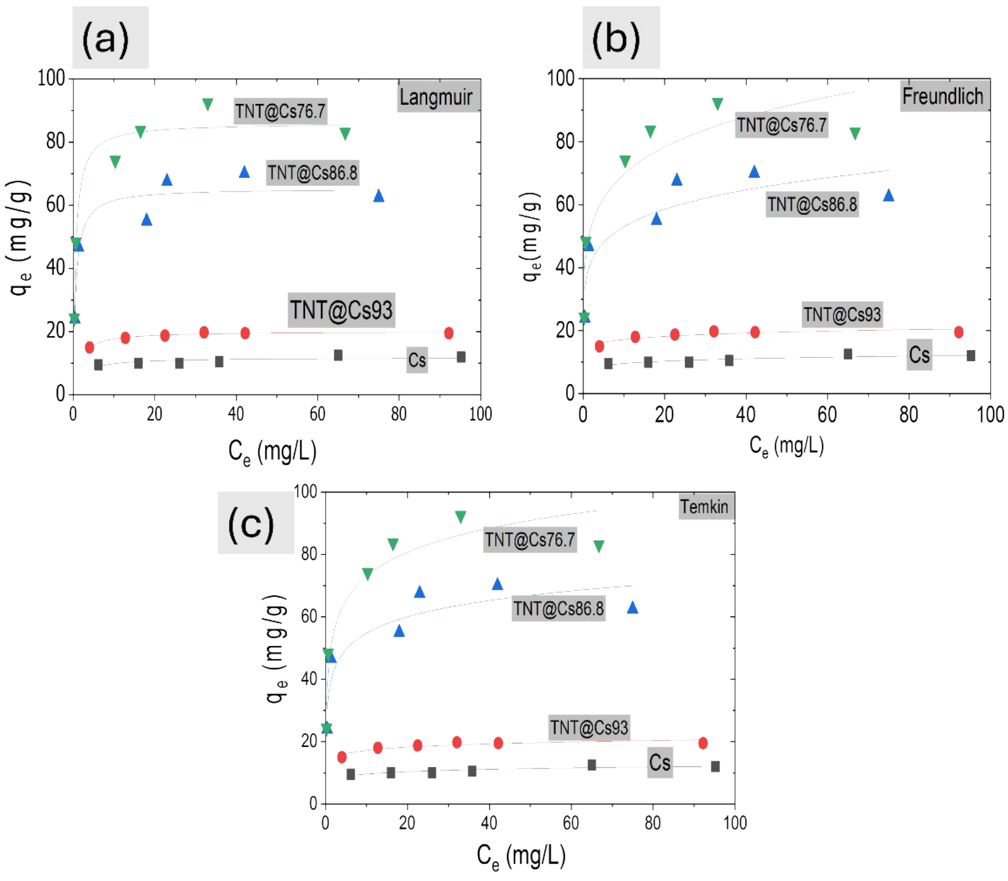

Figure S2: The non-linear isotherm curves for the (a) Langmuir, (b) Freundlich, and (c) Temkin models.

Table S2: The parameters estimated from the non-linear adsorption isotherm models for the adsorption of CV dye.

Adsorbent	Freundlish model			Langmuir model			Temkin model		
	K_f (mg/g)	n	R^2	K_L (L/mg)	q_m (mg/g)	R^2	b_t	A_T (L /g)	R^2
**Cs**	7.494	9.434	0.757	0.513	11.764	0.449	1.092	652.89	0.732
**TNTs@Cs$_{93}$**	14.27	12.43	0.750	0.737	20.063	0.971	1.518	8130.14	0.790
**TNTs@Cs$_{86.8}$**	38.08	6.96	0.746	1.612	65.345	0.892	7.565	139.140	0.805
**TNTs@Cs$_{76.7}$**	47.32	5.94	0.820	1.773	86.036	0.9513	11.148	69.568	0.897
